# Improving team coordination in primary-care settings via multifaceted team-based feedback: a non-randomised controlled trial study

**DOI:** 10.3399/BJGPO.2020.0185

**Published:** 2021-03-24

**Authors:** Sylvia J Hysong, Amber B Amspoker, Ashley M Hughes, Houston F Lester, Erica K Svojse, Kashif Khan, Praveen Mehta, Laura A Petersen

**Affiliations:** 1 Center of Innovations in Quality Effectiveness and Safety, Michael E DeBakey VA Medical Center, Houston, Texas, USA; 2 Department of Medicine - Section of Health Services Research, Baylor College of Medicine, Houston, Texas, USA; 3 Department of Biomedical and Health Information Sciences, University of Illinois Chicago, Chicago, Illinois, USA; 4 Department of Management, University of Mississippi, Oxford, Mississippi, USA; 5 Richmond VA Community-Based Outpatient Clinic, Richmond, Texas, USA; 6 VA Great Lakes Health Care System, Westchester, Illinois, USA

**Keywords:** primary health care, audit and feedback, debriefs, team coordination, learning health system, embedded partnership research, quality of health care

## Abstract

**Background:**

Coordination is critical to successful team-based health care. Most clinicians, however, are not trained in effective coordination or teamwork. Audit and feedback (A&F) could improve team coordination, if designed with teams in mind.

**Aim:**

The effectiveness of a multifaceted, A&F-plus-debrief intervention was tested to establish whether it improved coordination in primary care teams compared with controls.

**Design & setting:**

Case-control trial within US Veterans Health Administration medical centres.

**Method:**

Thirty-four primary care teams selected from four geographically distinct hospitals were compared with 34 administratively matched control teams. Intervention-arm teams received monthly A&F reports about key coordination behaviours and structured debriefings over 7 months. Control teams were followed exclusively via their clinical records. Outcome measures included a coordination composite and its component indicators (appointments starting on time, timely recall scheduling, emergency department utilisation, and electronic patient portal enrolment). Predictors included intervention arm, extent of exposure to intervention, and degree of multiple team membership (MTM).

**Results:**

Intervention teams did not significantly improve over control teams, even after adjusting for MTM. Follow-up analyses indicated cross-team variability in intervention fidelity; although all intervention teams received feedback reports, not all teams attended all debriefings. Compared with their respective baselines, teams with high debriefing exposure improved significantly. Teams with high debriefing exposure improved significantly more than teams with low exposure. Low exposure teams significantly increased patient portal enrolment.

**Conclusion:**

Team-based A&F, including adequate reflection time, can improve coordination; however, the effect is dose dependent. Consistency of debriefing appears more critical than proportion of team members attending a debriefing for ensuring implementation fidelity and effectiveness.

## How this fits in

A&F in health care has largely consisted of summaries or dashboards of clinical performance^1^ to individuals at varying levels of aggregation and little else. This study demonstrates reflection is an important component in the design of effective A&F and that, with proper exposure and adherence, it can be effective for improving non-clinical processes impacting quality of care.

## Introduction

Primary care is the gateway for most patients into the healthcare system, and care coordination is one of its most essential functions.^2^ Consequently, primary teams require effective team coordination to deliver effective care coordination; for example, for teams to produce successful outcomes, members must be able to effectively sequence and route interdependent clinical work so patients do not 'fall through the cracks'.^3^ Effective teamwork is not part of most clinicians’ training. Thus, effective tools designed for teams are needed to improve team coordination and, in turn, coordination of care.

Multiple frameworks seek to codify best practices for healthcare coordination. For example, the framework proposed by the Agency for Healthcare Research and Quality proposes specific coordination activities (for example, assessing needs and/or goals, facilitating transitions, follow-up), and broader approaches (for example, health information technology to facilitate coordination, a healthcare home for patients) that should facilitate coordinated care.^4^ However, the framework omits the fundamental processes and mechanisms behind successful coordination. Without understanding the 'how' of coordination, it is difficult to improve team coordination and, in turn, deliver higher quality care. Gittel’s theory of relational coordination^5^ begins to address this shortcoming, by positing that shared goals, shared knowledge and mutual respect between groups or teams promote frequent, timely, accurate, problem-solving communication and vice versa, allowing them to effectively coordinate their work.

Based on 30 years of coordination research, Okhuysen and Bechky’s^3^ integrated framework expands Gittel’s work to explain the underlying mechanisms of effective coordination, proposing three necessary, integrating conditions: predictability (knowing what tasks are involved and when they happen); accountability (clarity over who is responsible for what); and common understanding (a shared perspective on how each individual's work contributes to the whole). Together, they allow team members to collectively accomplish interdependent tasks, consistent with recent research.^6,7^ The framework also posits specific mechanisms, such as plans and rules, roles, and routines, which research shows make these integrating conditions possible, and which are associated with better coordination and, subsequently, better task performance.^8,9^

Using this framework, areas ripe for intervention could be identified and appropriate tools could be selected. Health care is highly protocolised, with many explicit policies, rules, and general routines in place. What is needed are tools that can help teams monitor their performance and adapt their processes flexibly to maintain predictability, accountability, and common understanding within the team. From this perspective, A&F is viable; A&F is effective for changing clinician behaviour, particularly when correct-solution information is presented.^10^ However, clinical work requiring more complex coordination is more difficult to improve.^11^ Thus, even when designed correctly, traditional feedback reports and dashboards may be insufficient to improve team-based outcomes dependent on coordination owing to the interdependent nature of the underlying work. From this perspective, team-based A&F may require additional elements targeted at improving predictability, accountability, and common understanding.

An important component of any A&F, whether team- or individual-focused, is the opportunity for reflection in order to create the conditions for behaviour change.^12^ Because team performance is driven by interactions among team members (team processes) in addition to individual member performance, more structured approaches to post-feedback reflection and correct-solution information are needed for teams than for individuals. Rudolph and colleagues^13^ advocate for debriefing as a form of formative assessment that can help those who debrief *'* *develop and integrate insights from direct experience into later action* *'*, particularly if the participants investigate the conditions underlying a performance gap. Reflection and debriefing can be especially useful when the members of a team serve on multiple teams, and their cognitive resources are spread thin. Interactive feedback elements, such as team debriefs, encourage such reflection and self-discovery, and explicitly elicit team-based opportunities for improvement, thus amplifying individual-level effects of A&F to the team.

### Study objective

The objective of this study was to test effectiveness of a team-based A&F-plus-debrief intervention at improving coordination in primary care teams. It was hypothesised improved team coordination with the intervention compared with matched administrative controls.

## Method

### Design

This controlled trial of a team-based, multifaceted A&F intervention is part of a larger partnered research study.^14^ Details of this partnership project and its methods appear elsewhere;^15^ guidelines of Hysong *et al* for reporting partnered research have been followed.^16,17^

### Partnership approach

This study was conducted in close partnership with the US Department of Veterans Affairs (VA) Great Lakes Healthcare system (Network 12), which has eight VA medical centres (VAMCs) and 38 community-based outpatient clinics; and the South Central VA Healthcare Network (Network 16), consisting of 10 VAMCs and 54 outpatient clinics. Experts in industrial and organisational psychology, primary care, data management and/or programming, and statistics, along with operational leaders and primary care clinicians, comprised the research team. The research team has long-standing relationships with both networks. For this study, the research team contributed scientific and methodological expertise and protected research effort; operational partners contributed study sites, data, and protected staff time for participation.

### Site and team selection

Operational partners helped select intervention-arm sites, based on resource availability, resulting in 34 teams at four sites: one large VAMC from Network 12 and three smaller outpatient clinics from Network 16, selected based on driving distance from the research team. To be eligible, teams were required to be led by an attending primary care provider; specialty and resident-led teams were excluded.

The methodology of Byrne and colleagues was applied, which matches peer groups for facilities based on site characteristics to identify control sites.^18^ The final 34 control teams were selected from five sites. The control and intervention teams were matched based on MTM (the average number of teams to which their members were assigned).^19^

### Participants

The members of 44 primary care teams (*n* = 83) were invited via email at four VA primary care clinics from Networks 12 and 16, consisting of primary care providers, registered nurse care managers, licensed vocational or practical nurses, and scheduling clerks. As the recommended core team size is four members, many team members are assigned to more than one team to ensure sufficient coverage. The authors followed up as needed via email, instant messaging, phone calls, and in person. For a given team to be enroled into the study, at least two of a team’s core members (provider, care manager, clinical associate, and clerical associate), must have enroled.

### Intervention

**Figure 1.**
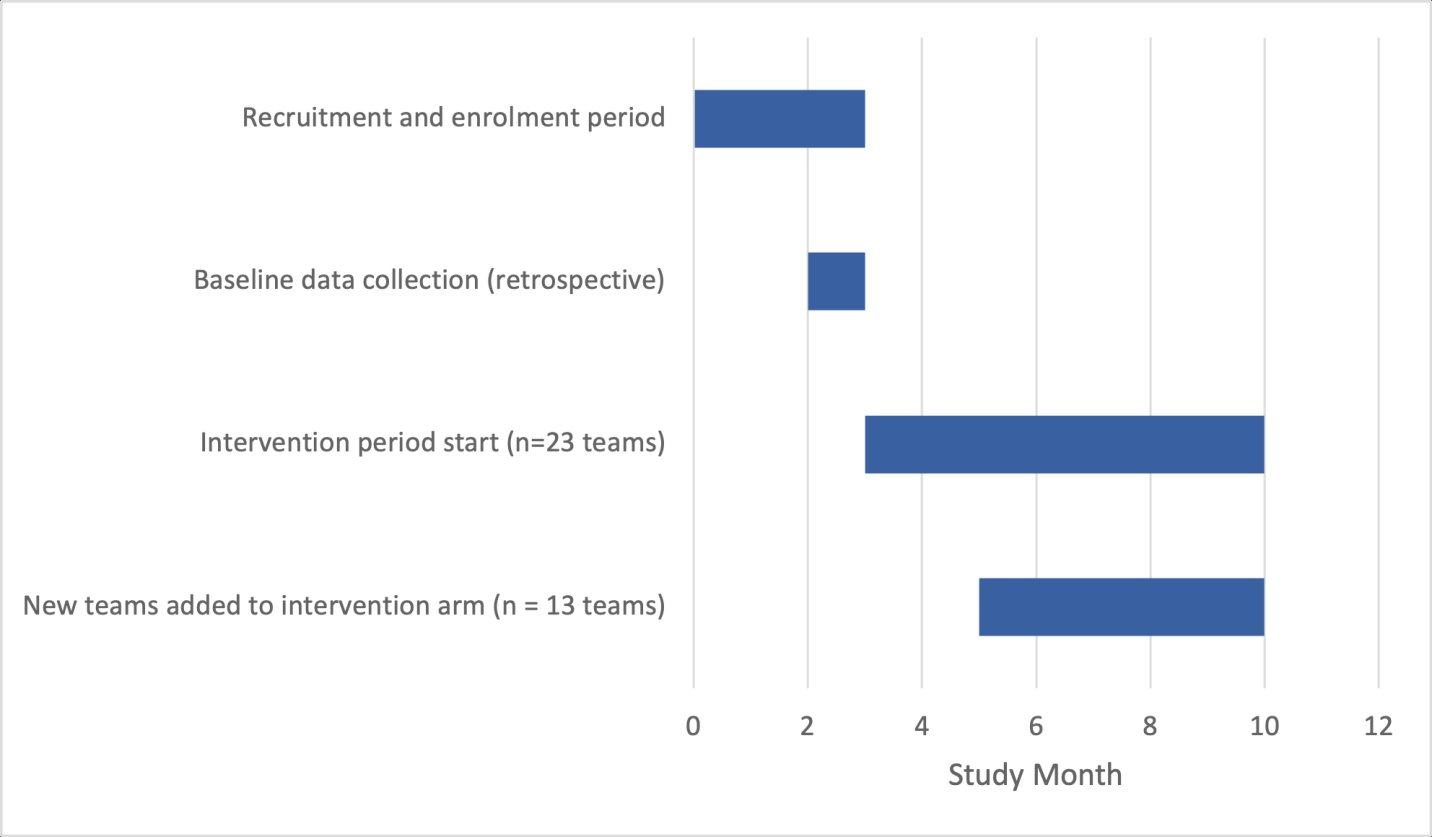
Team recruitment, enrolment, and intervention timeline

Consistent with research and best practice,^10,12,20^ the multifaceted, team-based intervention consisted of a one-time, 30-minute participant training followed by monthly feedback reports and debriefings over a 7-month period. Figure 1 details the enrolment and participation timeline for the teams.

#### Training

Participants received a 30-minute training webinar^21^ explaining the fundamentals of coordination (predictability, accountability, and common understanding), a step-by-step guide to interpreting their feedback report, and the mechanics of the team debriefs (described below).

#### Web-based A&F reports

**Figure 2.**
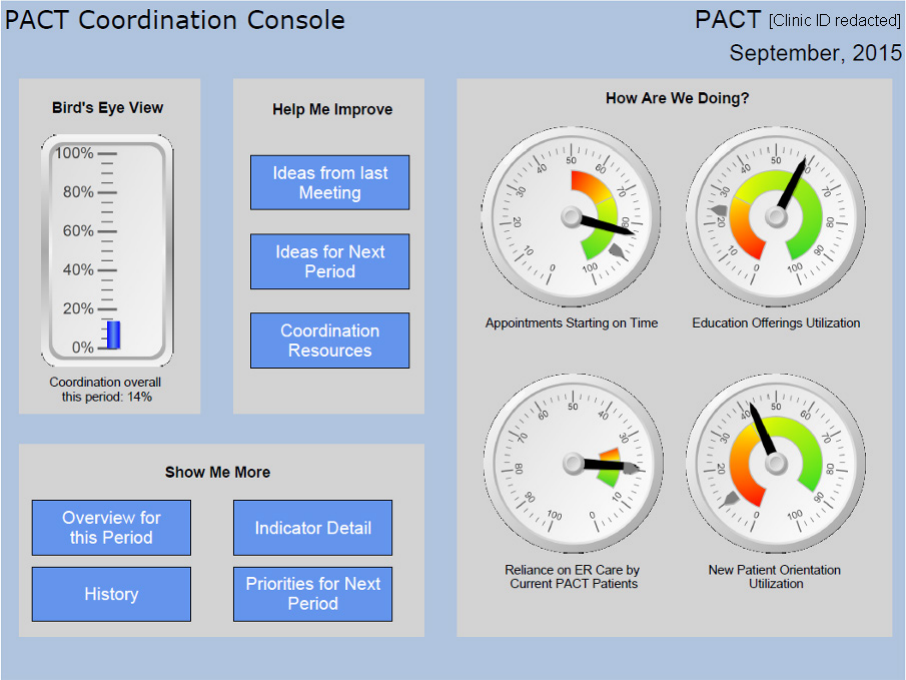
Screenshot of feedback report dashboard. ER = emergency room. PACT = Patient Aligned Care Team.

Participants received monthly feedback reports containing performance information on each measure of coordination and the composite (see Figure 2). The report format follows current recommendations for evidence-based feedback intervention design.^22^ Although designed for an interactive, web-based experience, the report can be used in print (as done by some teams). In addition to traditional evidence-based components,^10,23,24^ the report included novel, yet evidence-based features that distinguish it from currently available dashboards, such as functions that show incremental value gain per additional unit of performance improved, and prioritisation of indicators (tailored for each team at each time point), based on value-gain maximisation.

#### Team debriefs

One week after sending feedback reports, the research team held 15- to 20-minute debriefings with teams to facilitate guided discussions about coordination and next steps for improvement. Team debrief structures were adapted from the Team Dimensional Training approach for guided self-correction,^25,26^ with which members diagnose and solve their team’s performance problems with guidance as to topics they should discuss (in this case the feedback report guided the discussion) and ways to do so constructively.^27^ Facilitated by a research team member, clinical teams discussed the roles and needs of each team member to perform successfully on the feedback-report metrics as a set, facilitate coordination within the team and, ultimately, improve clinical performance. Importantly, by the end of each debrief, the team wrote two things to start, stop, and continue doing to improve coordination, as measured by report indicators; these were captured in a “start/stop/continue” (SSC) form. All debriefing materials (including the SSC) were available to teams post-debrief on the feedback-report website.

### Intervention fidelity

Dane and Schneider^28^ propose five properties to characterise fidelity: exposure (the extent to which participants received the intervention); adherence (the extent to which participants complete all aspects of the intervention as intended); quality of delivery (the extent to which the intervention is delivered to standard); participant responsiveness (participant reactions and satisfaction with intervention components); and programme differentiation (extent to which the intervention does not overlap or is confounded by similar initiatives). Under controlled research conditions, as is the case in this research study, most facets of fidelity can also be controlled, thereby mitigating any unintended impact on study results.

In this study the following were able to be controlled: the quality of delivery (feedback reports were generated and delivered via automated computer code; trained members of the research team delivered debriefings to the designed standard, confirmed by quality control recordings); adherence (feedback reports were reviewed and discussed during debriefs, and debrief facilitators [members of the research team] ensured SSC forms were completed at the end of the debrief); programme differentiation (the research team confirmed the selected sites and teams were not involved in similar initiatives during the duration of the study); and exposure to the feedback reports (reports were delivered to participants’ preferred email and printed copies were reviewed during debriefings). Although not controllable, participant responsiveness was also assessed through exit surveys at the end of the study, and exposure to the debrief component of the intervention by tracking attendance at team debriefs. Of all five facets, exposure to debriefs had the lowest extent of control and the highest potential for impacting findings. Therefore, attendance records were used to create measures of exposure to include in the analyses (see effect modifiers section, below).

### Measures

#### Dependent variable: coordination

To identify a suitable measure reflective of team coordination in primary care, an evidence-based methodology from industrial and organisational psychology called the Productivity Measurement and Enhancement System (ProMES) was relied on.^24,29^ Designed to develop performance measures, ProMES relies on a team of subject matter experts to determine the performance objectives of a unit (in this case coordination in primary care teams), generate new or identify existing performance measures that indicate the objectives are being accomplished, and develop 'contingencies', that is, prioritisation curves to help prioritise the set of performance measures according to gain or value to the organisation. Details of this process and the resulting objectives, indicators, and contingencies are published elsewhere.^15,30^ From seven measures identified by the experts as reflective of coordination in primary care teams, those available from existing data sources for all participating teams were employed: the percentage of (1) appointments that started on time; (2) recall appointments scheduled within 7 days of desired date (timely recall scheduling); (3) patients who used the emergency department (ED) for urgent or primary care complaints (ED utilisation); and (4) patients enroled in secure messaging through the electronic patient portal (electronic patient portal utilisation). These measures were then aggregated to calculate a composite measure of effectiveness.

#### Effect modifiers

##### Intervention exposure (fidelity)

To assess the impact of intervention exposure on study findings, three types of team-level exposure were calculated: (1) total exposure; (2) rate of exposure (number and percentage of intervention debriefings when at least one team member attended); and (3) strength of exposure (average percentage of team members attending debriefings per month). Conceptually, it was expected that the coordination of intervention teams with greater exposure to the intervention would improve more than that of teams with lower exposure. However, as the evidence is mixed on the impact of exposure on programme outcomes, the authors made no a priori hypotheses as to which operationalisation of exposure (rate, strength, or total exposure) would yield stronger effects.

##### Multiple team membership

Most team research assumes each member of a given team is assigned to one and only one team. In cases where team members are assigned to more than one team, the team member’s cognitive resources can be spread thin, thus hindering performance for all the teams involved.^19^ Having learnt during recruitment most clinicians indeed worked on multiple teams, data from the Team Assignments Report were extracted to calculate the average number of team memberships per person in each primary care team and better assess the impact of the intervention on the dependent variables of interest.

### Data analysis

#### Between-arm baseline differences

To ensure the intervention and control arms were comparable, independent samples *t-*tests were conducted for continuous variables and Fisher’s exact test was conducted for categorical variables to detect between-arm differences in each coordination measure (including the composite) and two team characteristics: team-level MTM, and clinical focus (primary care, infectious disease, or women’s health). Differences in MTM could unfairly disadvantage teams with higher MTM scores at baseline, while differences in the number of teams of a given type at baseline could unduly skew results toward one type of coordination measure versus another.

#### Tests of hypotheses

To test intervention effectiveness, five sets of linear growth-curve models were employed examining between-arm differences in improvement on each coordination measure, including the composite. For each coordination measure, a main-effects model was examined with time and intervention group as predictors.

#### Subgroup (effect modifier) analyses

To test the impact that MTM and the degree of exposure could have on the effect of the intervention on coordination, an interaction model was examined for each measure that included the main effects and the arm-by-time interaction. Simple slope analyses were also conducted examining the relationship between time and each coordination measure at high (+1 standard deviation [SD]) and low (-1 SD) levels of each predictor (see Table 1).^31^

**Table 1.** Number of team debriefings attended by one or more team members

Number of debriefings	Number of teams	Per cent of teams
1	6	17.65
2	3	8.82
3	17	50.00
4	2	5.88
5	3	8.82
6	1	2.94
7	2	5.88

Total exposure ranged from one to seven debriefings attended, with an average of 3.12 (SD = 1.57) and a median of 3. The rate of exposure ranged from 14.29% to 100%, with an average of 66.79% (SD = 30.18). The strength of exposure ranged from 6.25% to 41.50%, with an average of 19.13% (SD = 9.91). MTM in intervention teams ranged from 2.60 to 8.03 with an average of 5.20 (SD = 1.24).

**Table 3.** Results of multilevel growth curve models for each coordination measure^a^

	Individual coordination measures	Coordination composite^b^
Appointments starting on time	Timely recall scheduling	ED utilisation	Electronic patient portal enrollment
	*b* (SE)	*P*	*b* (SE)	*P*	*b* (SE)	*P*	*b* (SE)	*P*	*b* (SE)	*P*
Main-effects model										
Time	0.60 (0.30)	0.04	0.16 (0.71)	0.82	0.07 (0.08)	0.37	0.01 (0.05)	0.88	0.01 (0.003)	0.07
Study arm	-1.95 (1.29)	0.17	-1.71 (2.88)	0.57	-0.21 (0.16)	0.23	0.23 (0.08)	0.03	-0.02 (0.02)	0.33
Interaction model										
Time	0.55 (0.48)	0.26	0.14 (1.18)	0.91	0.09 (0.12)	0.47	-0.04 (0.09)	0.60	0.003 (0.004)	0.49
Study arm	-1.97 (1.33)	0.18	-1.72 (3.30)	0.62	-0.18 (0.21)	0.43	0.14 (0.14)	0.34	-0.02 (0.02)	0.27
Time x study arm interaction	0.08 (0.66)	0.90	0.02 (1.59)	0.99	-0.04 (0.17)	0.83	0.09 (0.11)	0.41	0.004 (0.01)	0.52

^a^All models control for multiple team membership and respective baseline of the coordination measure.^b^Combination of the four indicators (appointments starting on time, timely recall scheduling, ED utilisation, and electronic patient portal enrolment). ED = emergency department

All models (both main effects and interaction) included random intercepts and an autoregressive (ar[1]) covariance structure type. Three levels were included in the analysis: teams (level 2); nested within site (level 3); over time (level 1). All models controlled for the respective baseline of the coordination measure. Analyses were conducted with SAS (version 9.4).

## Results

### Participant enrolment

Fifty-seven members of 34 teams (54.8% of eligible teams) from four geographically distinct VA primary care clinics enroled in the study. The average within-team enrolment rate was 56.8%. The CONSORT diagram in Supplemental File 1 displays the recruitment flow and resulting participation.

### Between-arm baseline differences

MTM was significantly higher in the intervention arm than in the control arm, suggesting intervention members were spread more thinly than controls at baseline. Second, control teams exhibited significantly higher rates of electronic patient portal enrolment at baseline. No other characteristics significantly differed (see Table 2).

**Table 2.** Team characteristics, overall and intervention group differences

	Intervention(*n* = 34)	Control(*n* = 34)	*P* value^a^
Clinical focus, *n* (%)			0.81
Infectious disease	1 (2.94)	0 (0.00)	
Primary care only	18 (52.94)	17 (50.00)	
Women’s health	15 (44.12)	17 (50.00)	
Team-level multiple team membership, mean (SD)	5.20 (1.24)	4.16 (0.58)	<0.0001
Baseline measures			
Percentage of appointments starting on time	67.01 (24.50)	60.55 (25.49)	0.29
Percentage of timely recall scheduling	72.19 (17.80)	75.26 (25.70)	0.58
Percentage of ED utilisation	18.28 (8.61)	21.82 (11.32)	0.15
Percentage of electronic patient portal enrolment	22.43 (4.27)	29.05 (8.88)	<0.01
Coordination composite^b^	0.41 (0.14)	0.40 (0.18)	0.91

^a^Independent samples *t*-tests for all except team focus, which was a Fisher’s exact test.^b^Combination of the four indicators (appointments starting on time, timely recall scheduling, ED utilisation, and electronic patient portal enrolment). ED = emergency department

### Test of hypothesis: intervention effectiveness

No significant between arm differences in overall coordination (*b* = -0.02, *P* = 0.33) were observed. Individual indicator analyses showed both control and intervention arms significantly improved (*b* = 0.60, *P* = 0.04) in appointments starting on time. No other dependent variables significantly improved (Table 3).

### Subgroup (effect modifier) analyses

Given initial results revealed few between-arm differences in improvement, the authors proceeded with their planned effect-modifier analyses to rule out any potential interactive effects. Table 1 presents descriptive frequencies of the number and per cent of teams attending a given number of debriefings. Results of the simple slopes analyses are described below.

#### Rate of exposure

Rate of exposure significantly modified the effect of the intervention on overall coordination. Overall coordination significantly increased over time, yet only among teams with a greater (+1 SD) rate of exposure (*b* = 0.01, *P* = 0.002); teams with a lower (-1 SD) rate of exposure showed no change in overall coordination over time (*b* = -0.0001, *P* = 0.78).

#### Total exposure

Total exposure significantly modified the effect of the intervention on clinical reminder condition, ED utilisation, and patient enrolment in electronic patient portal secure messaging. Specifically, clinical reminder completion significantly increased over time, yet only among teams with greater (+1 SD) total exposure (*b* = 0.70, *P*<0.0001). Similarly, ED utilisation significantly decreased over time, yet only among teams with higher (-1 SD) total exposure (*b* = 0.28, *P*<0.0001). Conversely, secure messaging enrolment significantly increased over time, although only among teams with lower (-1 SD) total exposure (*b* = 0.13, *P* = 0.01); secure messaging enrolment did not significantly change over time (*b* = -0.04, *P* = 0.40) in teams with greater (+1 SD) total exposure.

#### Strength of exposure

Strength of exposure had no significant modifying effect on any coordination measure, including the composite.

#### Multiple team membership

MTM significantly modified the effect of the intervention on clinical reminder condition and patient enrolment in electronic patient portal secure messaging. Specifically, clinical reminder completion significantly increased over time, yet only among teams with lower (-1 SD) MTM (*b* = 0.84, *P*<0.0001). Conversely, secure messaging enrolment significantly increased over time, although only among teams with higher (+1 SD) MTM (b = 0.20, *P*<0.001); in teams with lower (-1 SD) MTM, however, significant enrolment decreases (*b* = -0.12, *P* = 0.004) were observed.

## Discussion

### Summary

This partnered research study tested the effectiveness of an A&F intervention at improving team coordination in primary care teams. Analyses indicated improvement in both control and intervention arms on two indicators (clinical reminder completion and ED utilisation), but no significant between-arm differences. In post hoc analyses within the intervention arm increases in electronic patient portal enrolment were found for teams attending relatively few debriefings or having relatively high MTM.

### Strengths and limitations

Strengths of the study included its partnership approach, which resulted in a more feasible and implementable intervention; and the use of electronic health record data for both A&F and study coordination measures, which meant more credible A&F and more reliable and valid coordination data. Further, the study also considers coordination from a process, rather than the traditional outcome perspective. That said, several limitations exist. First, three coordination measures required data unavailable administratively for control teams, precluding full comparison of all coordination indicators across arms. Second, team exposure to debriefings was inconsistent, despite protected time for participation. However, accounting for this statistically, it was observed that exposure modified the strength of the intervention’s impact on coordination, thus highlighting an important factor during implementation. Finally, changes in team process were not assessed, which may have helped better explain the observed improvements in coordination.

### Comparison with existing literature

The study's findings that a certain degree of intervention fidelity and minimum dose of exposure is necessary for improvements to occur is consistent with the ProMES literature. For example, in their meta-analysis of studies employing ProMES to improve performance across numerous industries including health care, Pritchard and colleagues^29^ found that on average, 10 feedback periods were needed to materially improve and sustain performance. Although the study only employed seven feedback periods, significant improvements were able to be observed, compared to baseline, in four of the five indicators of coordination when the number or per cent of feedback periods to which teams were exposed was taken into account. In general, the greater the exposure, the better the teams performed. This pattern is also consistent with feedback literature suggesting that feedback given with greater frequency (as opposed to once, or very infrequently) is also more likely to be effective.^10,20,23^

The finding that secure messaging enrolment increased for teams with higher MTM is counterintuitive. Several possible explanations exist. First, Pritchard and colleagues found that teams improved less after ProMES in cases where there was a high degree of prior feedback before the intervention, which is also consistent with the Cochrane review on A&F.^20^ As enrolment in secure messaging was a national priority for VHA, members of teams with high MTM would have had more opportunity to receive feedback than those with low MTM. Since the clerk, the role most commonly charged with helping patients enrol, is also the role in the team with the highest MTM (*M* = 3.2 teams, compared with providers *M* = 1.7 teams), then over time they will enrol more patients, thus explaining the unexpected direction of the moderation effect.

Pritchard and colleagues also found that the more closely the intervention followed the classic elements of ProMES (that is, higher intervention fidelity), the better the results. One important difference in the study from classic ProMES was the group receiving the debriefings. ProMES was originally designed for individual teams. However, contrary to patient-centred medical home recommendations, teams at the primary site exhibited high levels of MTM, with groups of teams behaving as a single clinic rather than as individual teams. To accommodate this structure, debriefings were conducted at the clinic level at this site, so as not to overburden individuals belonging to multiple teams, although feedback reports were still delivered at the individual team level. Although this formed an important component of the partnership approach, this deviation from classic ProMES may not be congruent with research suggesting that individualised feedback is more effective,^32^ thereby diluting results.

Finally, members of intervention-arm teams were assigned to more teams than members of control arm teams, making it more difficult to coordinate successfully and biasing results toward the null.

### Implications for research and practice

Multifaceted A&F can effectively improve selected aspects of coordination, but only if teams review their feedback and debrief consistently. Interestingly, the proportion of the team attending any given debriefing did not alter amount of improvement. As with any habit or learnt behaviour, the results suggest consistency of exposure to performance information and debriefing activity, even if the team is incomplete, is more critical to performance improvement than ensuring the entire team is present at a given debriefing.

A&F is often employed as an implementation strategy for other interventions.^33^ When A&F itself is the intervention, it requires implementation strategies of its own, especially in team settings. Future research should examine how traditional implementation strategies should be modified when (a) serving as interventions per se and (b) serving to help implement initiatives and interventions in teams.

## Trial registration number

ISRCTN15412521
